# Sustained three-year impact of a physical therapy quality improvement project in the medical intensive care unit

**DOI:** 10.1093/ptj/pzag100

**Published:** 2026-09-21

**Authors:** Megan A Watson, Ryan Peterson, Sarah E Jolley, William Niehaus, Kyle J Ridgeway

**Affiliations:** Department of Medicine, Division of Pulmonary, Allergy and Critical Care Sciences, University of Colorado, Aurora, CO 80045, United States; Carver College of Medicine, Department of Internal Medicine, University of Iowa, Iowa City, IA, United States; College of Public Health, Department of Biostatistics, University of Iowa, Iowa City, IA, United States; Department of Medicine, Division of Pulmonary, Allergy and Critical Care Sciences, University of Colorado, Aurora, CO 80045, United States; Department of Physical Medicine and Rehabilitation, University of Colorado, Aurora, CO, United States; Inpatient Rehabilitation Facilities, UCHealth University of Colorado Hospital and UCHealth, Aurora, CO, United States; Department of Physical Medicine and Rehabilitation, Physical Therapy Program, University of Colorado, Aurora, CO, United States

**Keywords:** Mobility, critical care, acute care, physical therapists, quality improvement

## Abstract

**Importance:**

Early rehabilitation and mobility are components of care bundles and clinical practice guidelines for patients who are critically ill. Improved delivery of physical therapy is associated with decreased intensive care unit (ICU) length of stay (LOS). Barriers exist to optimizing the delivery of physical therapy. Analyses of quality improvement (QI) are often narrow in scope. Care improvements may not persist. Opportunities remain to improve physical therapy delivery to patients in the medical ICU (MICU) while analyzing care delivery and ICU LOS.

**Objective:**

The objective was to determine whether a QI initiative sustained improvement in physical therapy delivery and associations with ICU LOS in the MICU.

**Design:**

This was a before–during–after comparative design.

**Setting:**

The study took place in the MICU at a quaternary, academic medical center.

**Participants:**

The study included 13,058 MICU admissions.

**Intervention(s):**

The intervention was a multifaceted physical therapy program including multidisciplinary education, physical therapists integrated into multidisciplinary rounds, automatic physical therapist consult orders, electronic health record hard stop for bedrest orders, and increased physical therapy staffing including a program coordinator physical therapist.

**Main Outcome(s):**

Our primary outcomes were delivery, timing, and frequency of physical therapy, as well as ICU LOS.

**Measure(s):**

Main outcomes were assessed by (1) odds of receiving physical therapy while in the MICU and (2) time from MICU admission to first physical therapist session and (3) ICU LOS.

**Results:**

During the intervention, an increased proportion of admits received physical therapy with a nearly 4-fold increase in the odds of receiving physical therapy. Time to first physical therapist session was decreased while the proportion of days with physical therapy in the ICU was increased. In adjusted analysis, ICU LOS decreased modestly. Findings were similar for 3 years post-QI.

**Conclusions:**

Despite variable resources and increased illness severity in the post-intervention period, durable improvements in the percentage of patients receiving physical therapy, odds of receiving physical therapy, time to physical therapy, and proportion of days with physical therapy occurred for 3 years post-QI. Secondarily, the during and post periods were associated with modest reductions in ICU LOS.

**Relevance:**

This work is applicable to the fields of: critical care rehabilitation, quality improvement, ICU mobility, and health care delivery.

## Introduction

Patients who are critically ill are at risk for acute multisystem sequelae and experience long term functional limitations.^1–3^ The physiology of critical illness, immobility, and treatment modalities commonly used in the intensive care unit (ICU), such as mechanical ventilation (MV), corticosteroids, and neuromuscular blockade, all contribute to increased morbidity among ICU survivors.^2^^,^^4–10^ The physical, cognitive, and psychological impairments, combined with the associated functional limitations survivors often experience for years, are collectively referred to as post-intensive care syndrome (PICS).^2^^,^^4^^,^^10–13^ Physical functional impairment is the most common PICS sequelae with some estimates indicating a prevalence up to 75% in ICU survivors.^3^^,^^8^^,^^14–18^

Patients who are critically ill and survivors of critical illness exhibit issues suggesting a significant need for rehabilitation including physical therapy.^7^^,^^19–21^ In the ICU, rehabilitation therapy and active mobility during critical illness are safe, feasible, and a component of clinical practice guidelines and care bundles. Specifically, enhanced rehabilitation and early mobilization, especially for patients requiring MV, are recommended in current clinical practice guidelines (Pain, Agitation/Sedation, Delirium, Immobility and Sleep Disruption; PADIS)^22^^,^^23^ and care bundles (A-F)^24^^,^^25^ supported by international societies.^22–28^ Involvement of physical therapists in the care of patients who are critically ill is associated with improved mobility practices and higher levels of patient mobility.^29–34^

While precise parameters on exact intervention types, timing, and dosages remain elusive,^35–37^ the impact of early rehabilitation, and active mobilization remain consistent topics of interest among researchers, clinicians, and hospital administrators aiming to optimize care delivery and improve a variety of outcomes.^38^^,^^39^ In aggregate, controlled studies, quality improvement (QI) initiatives, practice guidelines, and professional societies support providing physical therapy to more patients, earlier, and more often as a component of an overall strategy to enhance active patient mobilization.

However, there are significant barriers to delivering rehabilitation and increasing active mobilization among critically ill patients with low overall uptake of these recommended practices,^30^^,^^40–43^ especially among patients receiving MV via an endotracheal tube.^29^^,^^30^^,^^44–47^ Historically, physical therapists self-report less involvement in the care of patients in the medical ICU (MICU), compared to surgical or neurosurgical ICUs, with medical devices such as MV via endotracheal tube affecting intervention delivery.^46^^,^^48^ To improve the value of care, QI processes afford clinical, operational, and administrative stakeholders the opportunity to identify gaps in current practice and barriers to more ideal care delivery.^49^^,^^50^

QI focused on rehabilitation in critical care often strives to optimize the delivery of physical therapy by improving visit timing and frequency. Clinical-operational goals such as increasing the proportion of patients receiving therapy, initiating therapy earlier, and increasing therapy visit frequency in the ICU are important process metrics. Collectively, such improvements indicate not only an improved overall therapy dosage received by patients but often contribute to increased patient mobility.^31^^,^^32^

Length of stay (LOS) is an outcome of interest used in clinical trials, QI, and administrative analyses related to critical care rehabilitation and mobility.^38^^,^^51–54^ Improving the delivery of physical therapy and increasing active patient mobility are both consistently associated with decreased ICU LOS.^32^^,^^38^^,^^39^^,^^54–56^

Successful implementation of critical care rehabilitation appears multifactorial including such components as: physical therapist education and training, increased physical therapist staffing, therapy consultation order criteria, embedding therapists into critical care, program coordination, EHR optimizations, therapy and mobility pathways, and sedation protocols.^7^^,^^31^^,^^32^^,^^43^^,^^54^^,^^57–59^

Long-term analyses of critical care rehabilitation, such as physical therapy QI projects, are limited. Existing literature evaluates the impact of QI interventions over a period of months.^32^^,^^34^^,^^38^^,^^54^^,^^58^ It remains unknown if improvements in clinical practice such as the delivery of physical therapy and/or outcomes such as decreased LOS are sustained long term.^60^^,^^61^

This study investigates the delivery of physical therapy and associations with LOS in an academic MICU before, during, and 3 years after a physical therapy QI project. The clinical-operational aims of the program were to increase the proportion of patients receiving physical therapy, increase the frequency of physical therapy, and decrease the time from MICU admission to first physical therapist visit. This manuscript was prepared in accordance with the SQUIRE 2.0 guidelines for reporting QI work^62^ (Supplementary Material 1).

## Methods

### Objective

Our project sought to ascertain whether a 9-month mobility QI intervention resulted in sustained improvement over the following 3 years in delivery of physical therapy for MICU admissions as measured by: (1) the odds of receiving physical therapy while in the MICU, (2) the time from MICU admission to first physical therapist session, and (3) ICU LOS.

### Design

This study employed a before-during-after comparative design to assess the 9-month ICU mobility QI initiative. The QI program was conducted from April to December 2015, during which the below intervention was implemented. The study was categorized as a QI project and deemed exempt from full institutional review by the Colorado Institutional Review Board (IRB), as it did not involve human participant research.

Data were abstracted from the EHR in a standardized format^60^ using Health Data Compass, the clinical data warehouse for the University of Colorado Health System (UCHealth). Within the scope of the QI project and IRB approval, data were available for all MICU admissions from August 2011 through May 2019, allowing analysis of pre-, during-, and post-intervention periods. Data beyond May 2019 were not available due to subsequent changes in health system data governance and extraction procedures; analysis of later data would require additional IRB approval and a new data extraction request. We therefore focused on the longest continuous post-intervention period available.

### Setting

This QI occurred in the MICU at the University of Colorado Hospital (UCH) on the Anschutz Medical Campus within the UCHealth system. The UCH MICU is a 24-bed, closed ICU within an approximately 700 bed quaternary care academic hospital in the Denver Metropolitan Region. Detailed information on unit staffing is contained in Supplementary Material 2.

Prior to implementation and this investigation, a historic descriptive analysis (January 2012 through December 2014) of MICU admits with an ICU LOS of >2 days revealed that only 41% received physical therapy. On average, these patients received physical therapy on 39% of MICU days with the first physical therapist visit occurring 2.6 days (62.4 hours) after MICU admission.

### Participants

Our study included 13,058 admissions to the UCH MICU. This included 5924 admissions pre-intervention (August 2011–March 2015), 1389 admissions during the intervention (April 2015–December 2015), and 5745 admissions post-intervention (January 2016–May 2019). Fifty-one total admissions were excluded from the analysis for missing age at time of MICU admission.

### Intervention

The clinical-operational goals of the QI project were to (1) increase the proportion of MICU admissions receiving physical therapy, (2) decrease the time from MICU admission to first physical therapist session, and (3) increase the frequency of physical therapy in the MICU.

The multifaceted intervention included (1) the creation of a physical therapist program coordinator position to lead day-to-day care delivery, training, and program assessments, (2) increased physical therapy staffing from 0.5 full-time equivalent (FTE) on weekdays to 3 FTE Monday through Saturday, (3) automatic physical therapist consult orders for all MICU admissions, (4) hard stop and mandatory comments for any bedrest orders, and (5) the inclusion of physical therapists in daily MICU multidisciplinary rounds. Lastly, there was a significant (6) education and training component. Immediately prior to the intervention period, physical therapists were educated regarding safety guidelines that specifically focused on identifying patients who were not medically ready for active mobility. Physical therapists were trained on clinical logistics and communication strategies that were constructed collaboratively by the medical director, nursing leadership, and nurse educator of the MICU. Physical therapists participating in the MICU QI program received 24 total hours of critical care and ICU rehabilitation focused training, details of which can be found in Supplementary Material 2. Physical therapists also shadowed a MICU bedside nurse for 4 hours beginning at morning shift change.

Clinically, physical therapists prioritized evaluation for all MICU admissions who were requiring MV, had an extended ICU LOS (defined as >4–7 days) and/or exhibited physical functional impairment. Admissions with an anticipated ICU LOS less than 48 to 72 hours and/or minimal physical-functional impairment were not primary targets for physical therapy. Additional details regarding the intervention can be found in Supplementary Material 2.

Following the QI period, the components of the intervention were incorporated as a standard clinical program with routine and ad hoc assessments of clinical processes, staffing, clinical-operational process metrics, and outcomes such as length of stay. These trends were discussed by rehabilitation therapy administrators and clinical team members to identify barriers to current program goals and inform potential program modifications.

### Main outcome and measure

In the pre, during and post-intervention periods, we quantified performance on the 3 primary clinical-operational process metrics: percentage of MICU admissions receiving physical therapy; time from MICU admission to first physical therapist session; and frequency of physical therapist visits while in the MICU. Additionally, we performed more robust statistical investigations of the following outcomes of interest: (1) the odds of receiving physical therapy while in the MICU; (2) the time from MICU admission to first physical therapist session; and (3) ICU LOS.

### Statistical analysis

Baseline characteristics of the population were summarized both overall and by intervention period, with unadjusted associations within each intervention period. Differences between groups were evaluated using χ-square tests for categorical variables and Kruskal-Wallis tests for continuous variables. We summarized the receipt of physical therapy, hours from MICU admission to first physical therapist visit, and frequency of physical therapy while in the ICU as well as ICU and hospital LOS in hours.

For the time from MICU admission to first physical therapist session, we treated time to a patient’s first physical therapist session as a time-to-event outcome, right-censored at ICU discharge, with in-ICU death treated as a competing risk. This outcome is summarized with a cumulative incidence function and modeled with a Fine and Gray subdistribution hazard model.^63^ Additionally, a binomial-family logit-link generalized linear model (GLM) was used for receipt of physical therapy, and a negative binomial GLM for ICU LOS. The primary explanatory variable in all models was encoded as a categorical variable for intervention period: pre- (referent category), during-, and post-intervention. Models were adjusted for the patient-related factors of age categorized as: 18 to 39, 40 to 49, 50 to 59, 60 to 69, 70+ years, and self-identified sex of male or female. Sex is associated with care delivery and outcomes in critical care.^64^ Additionally, models were adjusted for receipt of MV (yes/no). Finally, a quantile regression model determined the effect of the intervention on different quantiles of ICU LOS. All analyses were conducted in R v4.4.0 (R Core Team).^65^

### Role of the funding source

The funder had no role in study design, data collection and analysis, decision to publish, or preparation of the manuscript.

## Results

### Demographics

Our analysis included 13,058 MICU admissions: 5924 pre, 1389 during, and 5745 post-intervention. Table 1 contains demographics and descriptive statistics. Across all periods, 55% of MICU admissions identified as male with 64% of the population greater than 50 years old. Compared to pre-intervention, a slightly greater percentage of admissions were in the older age groups in the during and post-periods. Notably, 35% of all admissions received MV. This percentage was statistically significantly different across the intervention periods, with a greater percentage of patients (40%) receiving MV in the post compared to pre-QI period (30%). There was also a significantly increased mean duration of MV in the during (1.9 days) and post-periods (2.2 days), compared to pre-intervention (1.7 days; *P* < .001). On average, 15% of patients died in the hospital. Mortality was higher in the during (15%) and post-periods (17%), compared to pre-intervention (13%).

**Table 1:** Descriptive statistics of study population*^a^*

Characteristic	Overall *n* = 13,058	Pre *n* = 5924	During *n* = 1389	Post *n* = 5745	*P*
**Sex**					.6
Female, *n* (%)	5911 (45%)	2691 (45%)	644 (46%)	2576 (45%)	
Male, *n* (%)	7147 (55%)	3233 (55%)	745 (54%)	3169 (55%)	
**Age range, *n* (%)**					<.001
18–39	2713 (21%)	1224 (21%)	266 (19%)	1223 (21%)	
40–49	1909 (15%)	928 (16%)	233 (17%)	748 (13%)	
50–59	2941 (23%)	1415 (24%)	302 (22%)	1224 (21%)	
60–69	2891 (22%)	1252 (21%)	324 (23%)	1315 (23%)	
70+	2604 (20%)	1105 (19%)	264 (19%)	1235 (21%)	
**Received any physical therapy, *n* (%)**	6049 (46%)	1694 (29%)	928 (67%)	3427 (60%)	<.001
No physical therapy received	7229	4341	471	2417	
**Hours from MICU admit to first physical therapist session (Hours [IQR])**	32 (18–61)	55 (24–114)	23 (16–40)	28 (17–49)	<.001
**Hours from MICU admit to physical therapist consult order entered in EHR (Hours [IQR])**	0 (0–11)	15 (0–74)	0 (0–1)	0 (0–1)	<.001
No physical therapist consult order	2967	2422	137	408	
**Received MV, *n* (%)**	4540 (35%)	1777 (30%)	465 (33%)	2298 (40%)	<.001
**Days on MV, *n* (%)**					<.001
0	8518 (65%)	4147 (70%)	924 (67%)	3447 (60%)	
1	784 (6.0%)	310 (5.2%)	90 (6.5%)	384 (6.7%)	
2	1111 (8.5%)	432 (7.3%)	102 (7.3%)	577 (10%)	
3	619 (4.7%)	232 (3.9%)	56 (4.0%)	331 (5.8%)	
4	390 (3.0%)	152 (2.6%)	39 (2.8%)	199 (3.5%)	
5–6	472 (3.6%)	188 (3.2%)	55 (4.0%)	229 (4.0%)	
7–8	324 (2.5%)	130 (2.2%)	34 (2.4%)	160 (2.8%)	
9–10	247 (1.9%)	101 (1.7%)	24 (1.7%)	122 (2.1%)	
11–15	265 (2.0%)	106 (1.8%)	28 (2.0%)	131 (2.3%)	
15+	328 (2.5%)	126 (2.1%)	37 (2.7%)	165 (2.9%)	
**ICU LOS (Hours [IQR])**	47 (25–96)	47 (24–96)	46 (24–92)	48 (25–97)	.091
**Total hospital LOS (Hours [IQR])**	156 (75–341)	148 (71–325)	149 (73–357)	166 (81–357)	<.001
**In-hospital mortality, *n* (%)**	1966 (15%)	777 (13%)	204 (15%)	985 (17%)	<.001

^a^ EHR = electronic health record; ICU = intensive care unit; LOS = length of stay; MICU = medical ICU; MV = mechanical ventilation.

### Clinical-operational performance

Overall, approximately half the population (*n* = 5829) received 1 or more physical therapist visits. Compared to pre-QI, a larger proportion of patients received therapy earlier and more often both during and after the QI initiative (Supplementary Material 3).

The proportion of admits receiving physical therapy increased from 29% pre-QI to 67% during and 60% post-QI. For those receiving physical therapy, the median proportion of ICU days with a physical therapist session for those who received therapy increased from 33% pre-QI to 67% during and 56% post-QI.

#### Statistical modeling

*Physical therapy care delivery and length of stay*

Using the cumulative incidence function, we find that the median time to first physical therapist session decreased from 182 hours (7.6 days) pre-QI to 32 hours (1.3 days) during and 46 hours (1.9 days) post, indicating meaningful and sustained reductions (Figure 1).

**Figure 1:**
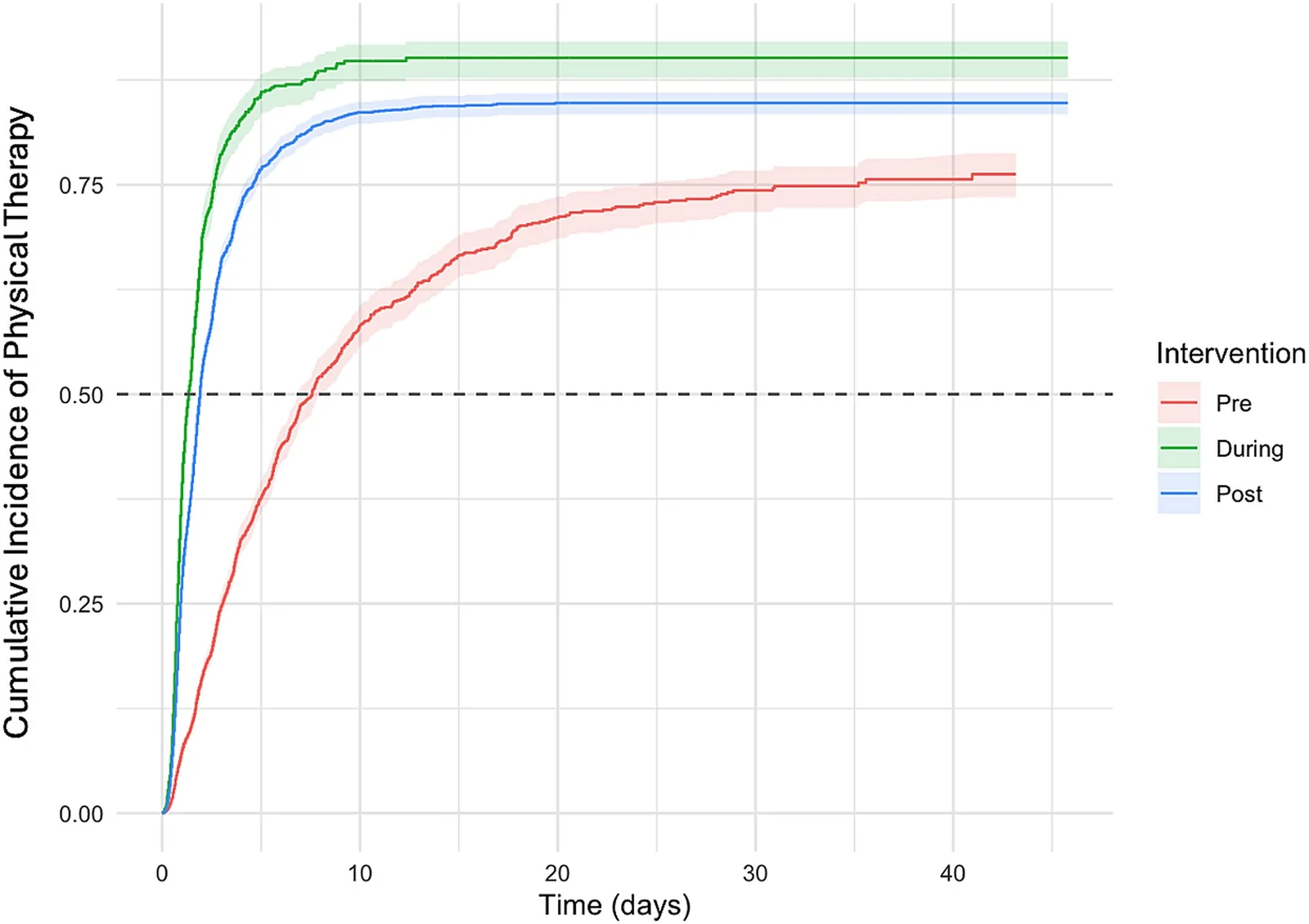
Cumulative Incidence Function Curves for Time from Unit Admission to First Physical Therapist Session.

Unadjusted logistic regression models (Table 2) demonstrated a 5-fold increase in odds of receiving physical therapy during the intervention, compared to pre-intervention (OR = 5.03; 95% CI = 4.44–5.70; *P* < .001). There was a similarly significant, albeit slightly less, increase in odds of receiving physical therapy in the post-period (OR = 3.69; 95% CI = 3.42–3.99; *P* < .001). After adjustment for sex, age and receipt of MV, there continued to be a 5-fold increase in odds of receiving physical therapy during the intervention (aOR = 5.19; 95% CI = 4.57–5.90; *P* < .001). Adjusted odds of receiving physical therapy in the post-period, compared to pre-intervention, was similarly increased (aOR = 3.64; 95% CI = 3.37–3.94; *P* < .001).

**Table 2:** Logistic regression model for receipt of physical therapy*^a^*

**Characteristic**	**Unadjusted**			**Adjusted**		
	OR	95% CI	*P*	OR	95% CI	*P*
**Intervention period**						
Pre	—	—		—	—	
During	5.03	4.44–5.70	<.001	5.19	4.57–5.90	<.001
Post	3.69	3.42–3.99	<.001	3.64	3.37–3.94	<.001
**Sex**						
Female	—	—		—	—	
Male	0.98	0.92–1.05	.6	0.96	0.89–1.04	.3
**Age range**						
18–39	—	—		—	—	
40–49	1.35	1.20–1.52	<.001	1.45	1.28–1.65	<.001
50–59	1.73	1.55–1.92	<.001	1.91	1.70–2.14	<.001
60–69	2.15	1.93–2.40	<.001	2.28	2.03–2.56	<.001
70+	2.35	2.10–2.63	<.001	2.55	2.27–2.87	<.001
**Received MV**						
No	—	—		—	—	
Yes	1.73	1.61–1.86	<.001	1.64	1.52–1.77	<.001

^a^ MV = mechanical ventilation.

In the Fine and Gray models (Table 3) adjusted for age, sex, and receipt of MV, the intervention period was associated with a shorter time to physical therapy (adjusted subdistribution hazard ratio [aSHR] = 4.61; 95% CI = 4.24–5.02; *P* < .001) compared to pre-intervention. This association persisted, but was attenuated, in the post-intervention period (aSHR = 3.23; 95% CI = 3.04–3.43; *P* < .001).

**Table 3:** Fine-gray subdistribution hazard model for time from unit admission to physical therapy (death during stay as competing risk)*^a^*

**Characteristic**	**Unadjusted**			**Adjusted**		
	**SHR**	**95% CI**	***P***	**SHR**	**95% CI**	***P***
**Intervention**						
Pre	—	—		—	—	
During	4.34	3.99–4.72	<.001	4.61	4.24–5.02	<.001
Post	2.97	2.81–3.15	<.001	3.23	3.04–3.43	<.001
**Sex**						
Female	—	—		—	—	
Male	0.99	0.94–1.04	.6	1.01	0.95–1.06	.8
**Age range**						
18–39	—	—		—	—	
40–49	1.09	1.00–1.20	.057	1.11	1.01–1.22	.033
50–59	1.21	1.12–1.32	<.001	1.28	1.18–1.39	<.001
60–69	1.38	1.27–1.49	<.001	1.36	1.25–1.48	<.001
70+	1.52	1.40–1.64	<.001	1.44	1.32–1.57	<.001
**Received MV**						
No	—	—		—	—	
Yes	0.62	0.58–0.65	<.001	0.53	0.50–0.56	<.001

^a^ MV = mechanical ventilation; SHR = subdistribution hazard ratio.

A negative binomial model was used to evaluate the impact of the intervention on ICU LOS (Table 4). In the unadjusted analysis, there was no difference in the mean ICU LOS between the during (MR = 0.97; 95% CI = 0.92–1.03) and post-periods (MR = 1.02; 95% CI = 0.99–1.06), compared to pre-intervention. After adjusting for age, sex and receipt of MV, there was a statistically significant 10% reduction in mean ICU LOS during the intervention, compared to pre-intervention (MR = 0.90; 95% CI = 0.86–0.95; *P* < .001). This finding was sustained in the post-intervention period, wherein the mean ICU LOS was 10% lower than pre-intervention (MR = 0.90; 95% CI = 0.88–0.93; *P* < .001).

**Table 4:** Negative binomial regression model for ICU length of stay*^a^*

**Characteristic**	**Unadjusted**			**Adjusted**		
	**Meanratio**	**95% CI**	***P***	**Meanratio**	**95% CI**	***P***
**Intervention**						
Pre	—	—		—	—	
During	0.97	0.92–1.03	.4	0.90	0.86–0.95	<.001
Post	1.02	0.99–1.06	.2	0.90	0.88–0.93	<.001
**Sex**						
Female	—	—		—	—	
Male	1.05	1.01–1.09	.006	1.01	0.98–1.04	.6
**Age range**						
18–39	—	—		—	—	
40–49	1.02	0.97–1.09	.4	1.08	1.03–1.14	.003
50–59	1.07	1.01–1.13	.012	1.12	1.07–1.17	<.001
60–69	1.14	1.08–1.20	<.001	1.18	1.13–1.24	<.001
70+	1.00	0.94–1.05	.9	1.10	1.05–1.15	<.001
**Received MV**						
No	—	—		—	—	
Yes	2.97	2.88–3.07	<.001	3.01	2.92–3.11	<.001

^a^ ICU = intensive care unit; MV = mechanical ventilation.

Similarly, quantile regression models (Supplementary Material 4; Figure 2) demonstrated an effect of the intervention greatest among those with the longest stays. For example, among patients in the 0.8 quantile (equivalent to an ICU LOS of 118 hours or approximately 5 days), the adjusted ICU LOS during the intervention, compared to pre-intervention, was reduced by 9 hours. For patients in the 0.9 quantile (8.6 days), ICU LOS decreased by 14 hours during and 16 hours post, compared to pre-intervention.

**Figure 2:**
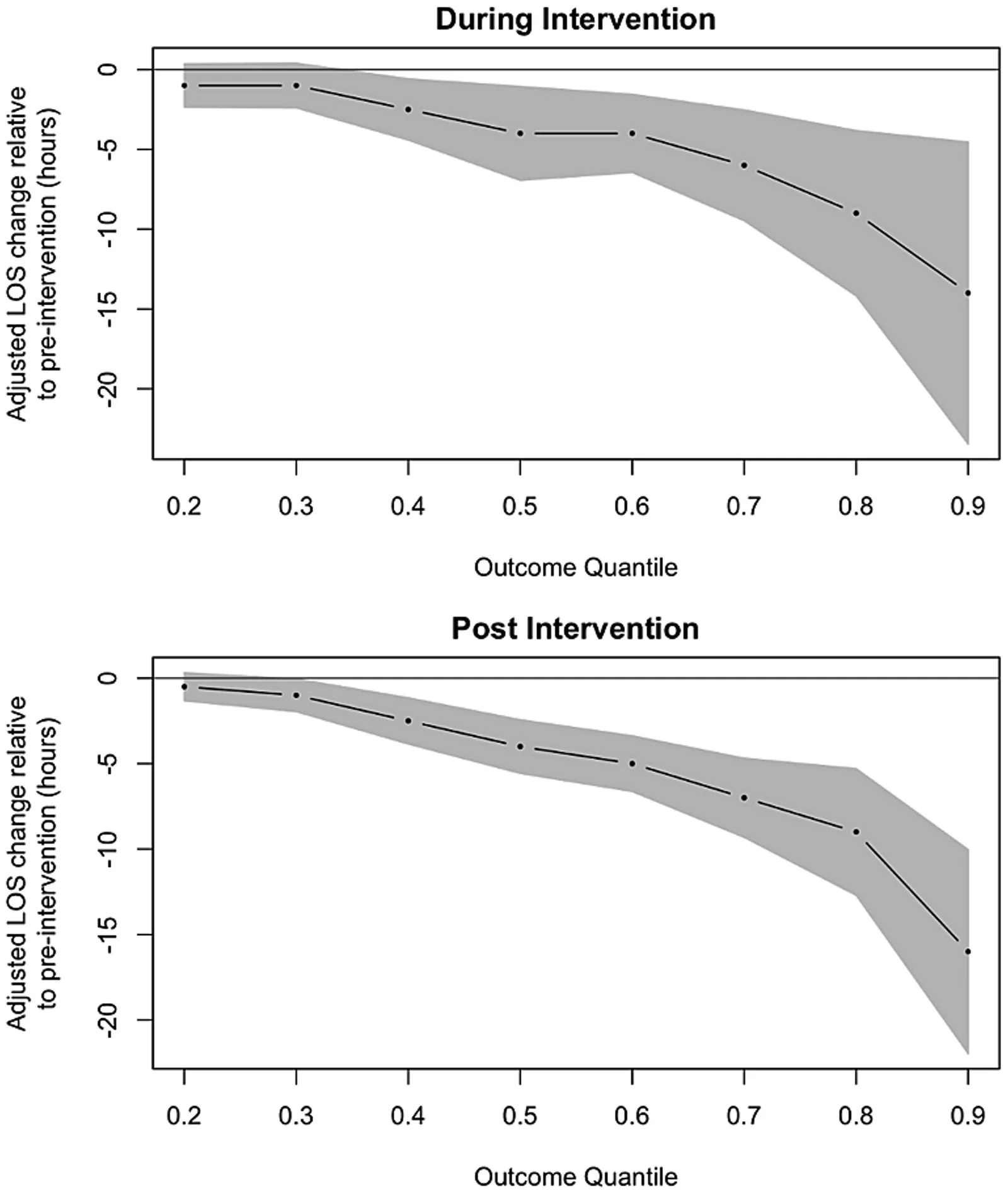
Quantile Regression Model for Adjusted Effects of the Intervention Period (Relative to Pre-Intervention) on Intensive Care Unit (ICU) Length of Stay (LOS) across Various Quantiles of ICU LOS.

## Discussion

In a QI interventional study, we analyzed the impact of a multifaceted QI strategy to increase physical therapy in the MICU of a single academic medical center including 5924 admissions pre-intervention, 1389 during, and 5745 post. Our results indicate dramatic improvements in the delivery of physical therapy for over 4 years (9-month QI intervention period plus 3 years and 4 months following) which was associated with modest decreases in ICU LOS.

Prior to the QI initiative, only 29% of admissions received their first session of physical therapy within a median of 182 hours (7.6 days). During the QI, 67% received physical therapy within a median of 32 hours (1.3 days), and for the 3 years after, 60% received physical therapy within a median of 46 hours (1.9 days). Median proportion of MICU days with physical therapy increased from 33% of days prior to 67% during and 56% after. In adjusted models, the odds of receiving physical therapy were 6-fold during the intervention period and nearly 4-fold post when compared to the pre-intervention period.

Our improvements in physical therapy care delivery are unique in magnitude, scope, and length of sustainment. For over 3 years after the QI, the clinical-operational aims of increasing the proportion of patients receiving physical therapy and improving the timing and frequency of sessions were all sustained. Statistically modeling upheld the descriptive findings. This long-term performance occurred in the context of demographic differences between time periods, most significantly in the post intervention period, and variable program resources in the 3 years post QI.

Specifically, patients in the post-intervention period were older with higher rates of death, mechanical ventilation, and mean duration of MV suggesting increased acuity of illness and patient complexity which could reasonably impact delivery of physical therapy. The presence of mechanical ventilation, especially via an endotracheal tube, is a well-established barrier to delivery of physical therapy. Lastly, around 50% of admits in all time periods exhibited a MICU LOS of 48 hours or less. Patients with a short ICU LOS were not primary clinical targets for physical therapy and were likely more difficult to reliably deliver physical therapy to while in the ICU. Further, it’s reasonable to assume they are less likely to benefit from ICU-based physical therapy.

To our knowledge, only 1 MICU physical therapy program demonstrated sustained improvements in physical therapy delivery over the course of years following a QI initiative.^31^ The analysis involved a much smaller cohort of patients (*n* = 120 pre; *n* = 123 post) admitted to a MICU with a diagnosis of acute lung injury. In the 3-year period analyzed, 89% of patients received therapy with a median time to therapy of 4 days. The median proportion of ICU days with therapy was 50%. Our study demonstrates similar findings in a dramatically larger, heterogenous cohort inclusive of *all* MICU admissions. Our results suggest the delivery of physical therapy can be improved for a general population of patients in the MICU while the observed associations with decreased ICU LOS may also apply to the broader MICU population. Importantly, these findings are consistent with prior feasibility studies,^66^ programs,^34^ and guideline recommendations supporting standardized delivery of rehabilitation therapy to improve the timing and frequency of therapy.^23^^,^^27^

In aggregate, our results suggest a multi-faceted strategy is likely an important mechanism to improve the delivery, timing, and frequency of physical therapy to patients in the MICU long-term while potentially decreasing ICU LOS. Implementation science, quality and process improvement methodologies, and specific mobility promotion frameworks support the need for systematic, multifaceted strategies to change care delivery.^7^^,^^20^^,^^58^^,^^67–70^ It’s worth highlighting our program contained EHR optimizations, multidisciplinary education and training, routine data feedback on care delivery performance, and increased clinical and administrative resources during *and* after the QI.

Compared to pre-QI, the odds of receiving physical therapy and time to first physical therapist session were significantly improved both during and post-QI; however, the magnitude of effect was less in the post-intervention period. This illustrates potential creep towards pre-QI performance questioning the sustainability of the intervention beyond 3 years. In addition to demographic differences between time periods, we anecdotally believe this is partially explained by resource allocation and prioritization. Daily, overall rehabilitation therapy department staffing was decreased by 15% in 2017 with a progressive decrease in overall MICU physical therapy staffing. In 2018, the coordinator role was eliminated. Further, patient readiness for physical therapy varies on a case-by-case basis. The slight decrease between the intervention and post-intervention periods, compared to pre-intervention, may reflect the differences in medical acuity combined with post QI resources. There was variability in staffing, administrative support, and the coordinator position.

Ongoing institutional prioritization, data feedback on care delivery performance, consistent physical therapy staffing, and administrative resources are likely required for long-term sustainability. For example, during the 3 years after the QI, the MICU physical therapy program daily staffing targets and actual daily staffing both varied. The creation and monitoring of minimum staffing levels, with triggers for increased (or decreased) daily staffing, may ensure reliable delivery of therapy in the long term. Further, not every MICU admission requires or may benefit from physical therapy. The precise proportion of admissions that *should* receive physical therapy, and the acceptable variance over time, is unknown.

The quantile regression model indicated the impact of the intervention on ICU LOS was proportional to ICU LOS. For example, admissions in the 0.7 quantile, whose ICU LOS ranged from 81 to 117 hours (3.4 to 4.9 days), saw a decrease in ICU LOS of 6 hours in the during- and 7 hours in the post-, compared to pre-intervention periods. Comparatively, those in the 0.9 quantile whose ICU LOS exceeded 207 hours (8.6 days), saw a greater than 14-hour reduction in ICU LOS in the during- and 16-hour reduction in the post-, compared to pre-intervention periods. Generally, our study observed a 6% to 7% reduction in ICU LOS for admissions in both quantiles illustrating a similar magnitude of relative ICU LOS decreases across varied durations of hospitalization.

While statistically significant, ICU LOS reductions of 6 to 16 hours may represent limited clinical impact at the individual patient level. However, at the institutional level, even small reductions in LOS are often considered meaningful, as they can substantially influence throughput and cost.^51^^,^^71^ Increasing illness severity over time likely obscured unadjusted ICU LOS comparisons. Adjustment for receipt of MV and patient characteristics revealed modest associations that were otherwise masked by case-mix changes.

Overall, our study illustrates that a multifaceted strategy was associated with improved delivery of physical therapy to patients admitted to a MICU, with durable practice changes observed for over 3 years following a QI intervention. However, while substantial improvements in the proportion of admits receiving therapy, time to first therapy, and frequency of therapy were observed, practice changes were not as dramatic in the post-intervention period. Secondarily, adjusted analyses demonstrated modest associations with decreased ICU LOS. These findings occurred in the context of increased patient acuity as well as variable and decreasing resources in the post-QI period. Importantly, the strategy included targeted tactics such as integration of physical therapists into the MICU, multidisciplinary education, EHR optimizations, resource prioritization, clearly defined clinical-operational aims (process measures), and routine data feedback on care delivery performance.

### Limitations

Our study brings novel information to the existing literature but is not without limitations. First, the QI initiative took place in a single medical ICU at 1 academic medical center, therefore the results are not broadly generalizable. As with all single-unit QI studies without a concurrent control unit, we cannot definitively rule out other contributions to the observed results. Specifically, we were unable to control for hospital-wide mobility practices, rehabilitation therapy practice, and MICU culture. Of note, no formalized hospital-wide mobility initiatives or process changes were implemented during the study period. Physical therapy delivery for the MICU was specifically designed and monitored throughout the study period. Given the decrease in therapy services across the hospital and a reduction in specific MICU program resources post-intervention it is unlikely these changes contributed to our positive results. Lastly, we cannot account for informal changes in MICU culture that may have evolved independently, or as a secondary effect, of the formal intervention components.

Further, we infer that the initiative impacted delivery of physical therapy which subsequently contributed to reductions in LOS, but given the retrospective nature, we cannot establish a causal relationship. Similarly, we were unable to account for additional demographic or clinical variables that may have impacted the delivery of physical therapy and/or LOS, such as admitting diagnosis, baseline functional status, medical prognosis, vasopressor requirements, need for renal replacement therapy, type and dosage of sedation, and use of neuromuscular blockade.

Data were only available from 2011 to 2019 which may limit current applicability. We are unable to quantify physical therapy delivery beyond 2019. Since 2019, and post-COVID-19 pandemic, the context of health care has evolved. However, studies during the pandemic indicate poor delivery of care bundles, rehabilitation, and mobility within critical care.^72^^,^^73^ Notably, COVID-19 infection status was not associated with implementation failure.^73^ We contend our strategy to address delivery of physical therapy to patients in the MICU is as applicable now as in 2019.^19^

We noted a secular trend toward increased illness severity that was reflected in increasing rates of MV across the intervention periods (30% pre-, 34% during-, and 40% post-intervention). We adjusted for this in our models by including MV status as a confounder; the association between intervention period and ICU LOS was attenuated in unadjusted analyses, likely reflecting increasing illness severity over time. We do not believe that the increase in MV rates is a direct effect of the physical therapy program, as receipt of MV typically occurs early in the ICU stay and therefore likely precedes receipt of physical therapy. However, we did not collect pertinent data on the timing of MV which would have allowed us to account for this in our analysis. Therefore, future studies should prospectively collect MV timing data to better distinguish illness severity from potential intervention effects on ventilation practice.

The lack of objective measures of physical function and mobility performance achieved are another limitation. Prior to 2015, physical function and mobility performance were not systematically measured nor documented in the electronic health record (EHR) by physical therapists or other disciplines such as nurses. Additionally, our institution did not capture multidisciplinary, standard measures of function or mobility for *all* patients. Thus, we were unable to quantify functional status or mobility performance within and between time periods. Similarly, we were unable to analyze change over time at the individual patient level. Future work should ensure the standard measurement of physical function and mobility performed.^67^^,^^74^ There is a need to directly study the impact of rehabilitation and mobility interventions on outcomes such as functional status, the highest level of mobility achieved during therapy sessions, and overall multidisciplinary mobility dosage: timing, frequency, and highest level achieved. Ideally, analyses should account for patients’ baseline physical function and other clinical factors.

Our observed reductions in ICU LOS were modest, ranging from 6 to 16 hours (6%-12% decrease). Although prior research and QI initiatives consistently demonstrate associations between improved physical therapy delivery and shorter ICU LOS, we were unable to control for all potential confounding factors nor establish causality. However, our models did adjust for key patient level characteristics including age, sex, and receipt of MV.

Lastly, it is crucial to consider the multifaceted, multicomponent, multidisciplinary nature of this intervention which included increased staffing, multidisciplinary education and training, EHR optimizations, and performance metric feedback. We are unable to ascertain if specific components were associated with our observed results. We were also unable to quantify specific resource changes over time such as precise staffing numbers, administrative support, and program prioritization. However, a multi-component approach is likely necessary to optimize care delivery, maximize impact, and ensure sustainability in a complex environment such as critical care.^20^^,^^31^^,^^58^^,^^61^^,^^67^^,^^68^ The necessary and sufficient components remain unknown, and future implementation studies should aim to isolate the contribution of individual intervention elements.

### Recommendations

Ongoing efforts to implement, and sustain, critical care rehabilitation and active mobility may be helped by the multi-faceted strategy investigated in this QI study. Our results, and experiences, suggest the importance of integrating physical therapists into day-to-day unit logistics, optimizing the EHR, creating specific clinical-operational goals, monitoring performance metrics, delivering data feedback, and performing multidisciplinary education and training. Further, the role of the physical therapist and importance of active mobility in the ICU should be integrated into training for other members of the multidisciplinary team such as nurses, respiratory therapists, and physicians. Such an approach helps mitigate known barriers, aligns with other successful programs^20^^,^^58^^,^^67^^,^^68^ and leverages QI recommendations. Measurement of clinical-operational performance delivering therapy, quantification of patient mobility performance, and systematic measurement of physical function will improve the ability to understand clinical operations, improve care, and perform robust research.^60^^,^^61^^,^^75–77^ This strategy aligns with quality and process improvement, implementation science, and learning health system frameworks as well as other successful approaches to optimizing delivery of rehabilitation therapy and increasing patient mobility.^59^^,^^67^^,^^74^ We believe these actions can contribute to improved rehabilitation, increased active mobilization of patients who are critical ill, and ultimately optimized outcomes.

## Supplementary Material

PTJ-2025-0576_R2_Supplementary_Material_pzag100

## Data Availability

Data that support these findings are available from the corresponding author upon reasonable request.
