# Supplementary material for: Sustained three-year impact of a physical therapy quality improvement project in the medical intensive care unit
Source: Phys Ther. 2026 Sep 21;106(10):pzag100. doi: 10.1093/ptj/pzag100 (PMC13720727; doi:10.1093/ptj/pzag100)
Supplement: Supplementary file 1 [file ptj-2025-0576_r2_supplementary_material_pzag100.pdf]

Supplementary Material 1

Research and reporting methodology
Revised **Standards for QUality Improvement Reporting Excellence** (**SQUIRE 2.0**) publication guidelines

Notes to authors

- The SQUIRE guidelines provide a framework for reporting new knowledge about how to improve healthcare.
- The SQUIRE guidelines are intended for reports that describe system level work to improve the quality, safety and value of healthcare, and used methods to establish that observed outcomes were due to the intervention(s).
- A range of approaches exists for improving healthcare. SQUIRE may be adapted for reporting any of these.
- Authors should consider every SQUIRE item, but it may be inappropriate or unnecessary to include every SQUIRE element in a particular manuscript.
- The SQUIRE glossary contains definitions of many of the key words in SQUIRE.
- The explanation and elaboration document provides specific examples of well-written SQUIRE items and an in-depth explanation of each item.
- Please cite SQUIRE when it is used to write a manuscript.

**Text section and item name**	**Page/line no(s).**
	**info is located**
**Title and abstract**	
1. **Title**	
Indicate that the manuscript concerns an initiative to improve healthcare (broadly defined to include the quality, safety, effectiveness, patient-centredness, timeliness, cost, efficiency and equity of healthcare).	1
2. **Abstract**	
a. Provide adequate information to aid in searching and indexing.	4 (line 7-8)
b. Summarise all key information from various sections of the text using the abstract format of the intended publication or a structured summary such as: background, local problem, methods, interventions, results, conclusions.	2-4
**Introduction: Why did you start?**	
3. **Problem description** - Nature and significance of the local problem.	9 (line 19) – 10 (line 2)
4. **Available knowledge** - Summary of what is currently known about the problem, including relevant previous studies.	3-8

5. **Rationale** - Informal or formal frameworks, models, concepts and/or theories used to explain the problem, any reasons or assumptions that were used to develop the intervention(s) and reasons why the intervention(s) was expected to work	6 (line 19) – 7 (line 19)
6. **Specific aims** - Purpose of the project and of this report.	8 (lines 11-14)
**Methods: What did you do?**	
7. **Context** - Contextual elements considered important at the outset of introducing the intervention(s).	9 (lines 13-17)

8. **Intervention(s)**	
a. Description of the intervention(s) in sufficient detail that others could reproduce it.	10 (line 12) – 11; Supplementary Material 2
b. Specifics of the team involved in the work.	10 (line 12) – 11; Supplementary Material 2
9. **Study of the intervention(s)**	
a. Approach chosen for assessing the impact of the intervention(s).	12 (lines 1-8)
b. Approach used to establish whether the observed outcomes were due to the intervention(s).	12 (lines 1-8)
10. **Measures**	
a. Measures chosen for studying processes and outcomes of the intervention(s), including rationale for choosing them, their operational definitions and their validity and reliability.	12 (lines 1-8)
b. Description of the approach to the ongoing assessment of contextual elements that contributed to the success, failure, efficiency and cost.	11 (lines 17-21)
c. Methods employed for assessing completeness and accuracy of data.	10 (lines 8-9)
11. **Analysis**	
a. Qualitative and quantitative methods used to draw inferences from the data.	12 (line 11) – 13 (line 8)
b. Methods for understanding variation within the data, including the effects of time as a variable.	12 (line 11) – 13 (line 8)
12. **Ethical considerations** - Ethical aspects of implementing and studying the intervention(s) and how they were addressed, including, but not limited to, formal ethics review and potential conflict(s) of interest.	8 (lines 19-21)
**Results: What did you find?**	
13. **Results**	
a. Initial steps of the intervention(s) and their evolution over time (eg, time-line diagram, flow chart or table), including modifications made to the intervention during the project.	Supplementary Material 2
b. Details of the process measures and outcomes.	13-16
c. Contextual elements that interacted with the intervention(s).	13-16
d. Observed associations between outcomes, interventions and relevant contextual elements.	13-16
e. Unintended consequences such as unexpected benefits, problems, failures or costs associated with the intervention(s).	19 (line 20-21)
f. Details about missing data.	10 (line 8-9)

**Discussion: What does it mean?**	
14. **Summary**	
a. Key findings, including relevance to the rationale and specific aims.	16 (lines 11-16
b. Particular strengths of the project.	18 (lines 2-14)
15. **Interpretation**	
a. Nature of the association between the intervention(s) and the outcomes.	18 (line 16) – 19 (line 2)
b. Comparison of results with findings from other publications.	18 (lines 2-14)
c. Impact of the project on people and systems.	21 (lines 1-12)
d. Reasons for any differences between observed and anticipated outcomes, including the influence of context.	21 (lines 7-12)
e. Costs and strategic trade-offs, including opportunity costs.	Supplementary Material 2
16. **Limitations**	
a. Limits to the generalisability of the work.	21-24
b. Factors that might have limited internal validity such as confounding, bias or imprecision in the design, methods, measurement or analysis.	21-24
c. Efforts made to minimise and adjust for limitations.	21-24
**Conclusions**	
a. Usefulness of the work.	25
b. Sustainability.	25
c. Potential for spread to other contexts.	21 (line 15) – 22 (line 5)
d. Implications for practice and for further study in the field.	23 (line 21) – 24 (line 3)
e. Suggested next steps.	23 (line 21) – 24 (line 3)
**Other information**	
18. **Funding** - Sources of funding that supported this work. Role, if any, of the funding organisation in the design, implementation, interpretation and reporting.	26 (line 21)

Ogrinc G, et al. BMJ Qual Saf 2015;0:1–7. doi:10.1136/bmjqs-2015-004411
Downloaded from http://qualitysafety.bmj.com/ on January 2, 2017

Supplementary Material 2

Medical ICU PT Program Components

Item	Pre	During	Post	Comments
Coordinator or clinical lead?	N	Yes, 0.5 FTE	Yes, 0.25 FTE	Post: Gradual decrease in administrative/lead time with eventual phase out of role
Weekday (M-F) Staffing: Full Time Equivalent Physical Therapists (average)	0.5	3	2.0-2.5	Post: Progressive decrease and variable staffing in PT
Weekend Staffing	0	1 Saturday<br>0 Sunday	0-1 Saturday<br>0 Sunday	Post: eventual removal of weekend staffing
EHR Optimizations	No	PT auto-consulted as part of MICU admission order set<br><br>Hard stop on all bedrest orders with required comment. Modified language to avoid bedrest orders	Same as during	
Formal MICU PT Training?	No	Yes	Variable	Progressive decrease in training time for new PTs. Training for MICU integrated into overall critical care training procedure

Interdisciplinary education and engagement on MICU PT?	No	Yes	No	
Specific clinical-operational goals and process metrics?	No	Yes	Yes	Key performance indicators for all admits and specifically for those requiring MV:<br>1) % of admits receiving PT<br>2) Time to first PT visit<br>3) % of MICU days with PT visit
Target Patient Populations	No, dependent on individual therapist assessments	Yes<br>All medical ICU admits with particular focus on patients requiring mechanical ventilation	Yes<br>Patients with physical, functional impairment, requiring mechanical ventilation, and specialty populations (high pulmonary acuity, pre-lung transplant)	Patients requiring MV always a focus during and post.
Data Feedback on process metrics / key performance indicators and outcomes?	n/a	Quarterly to PT team, pulm & crit care providers, MICU RN leadership	Every 1-3 months to PT team	Post: Gradual decrease in data feedback with eventual phase out

Education and Training

Topic	Total Time	Instructor	Details

ICU Rehab: Background and Principles	12 hours (6 sessions, 2 hours each)	QI Program Coordinator, Physical Therapist with ICU clinical and research experience	1) Outcomes and Sequalae of Critical Care<br>2) Safety and Feasibility of Rehab and Mobility<br>3) Standard Clinical Examination<br>4) Functional Assessment & Measures<br>5) Treatment Approach & Considerations: Focus on active mobilization, functional progression, and response dependent progression<br>6) Psycho-social components, communication, and collaboration
Critical Care and Medical ICU Basics	4 Hours	Medical ICU Nurse Educator	1) Lines and Physiologic Monitoring<br>2) Medications<br>3) Diagnoses, Medical Management, and Medical Procedures
Mechanical Ventilation and Respiratory Devices	4 hours	Respiratory Therapist, Critical Care Specialist	1) Mechanical Ventilation: Indications and Function<br>2) Heated High Flow

			3) Ventilator Liberation: Research and Protocols
Nurse Shadowing	4 hours	Clinical Nurse	1) Observation of nursing workflows<br>2) Discussion on PT program and impact of mobility<br>3) Therapy and mobilization as indicated

Physical Therapy "Red light" Exclusion Criteria

Ruling out patients not ready for physical therapy

1) End of life designation, focus on comfort care, and/or hospice?
2) Neuromuscular blocking agents **OR** ordered for deep sedation (RASS ≤ -3) to treat a medical condition, for which it would be unsafe to awaken the patient?
3) Actively and acutely bleeding with unstable hemoglobin and hematocrit?
4) Trend of increasing mechanical ventilation support **OR** FiO2 >0.8 and/or PEEP >12?
5) Increasing/up titration of vaso-active medication dosage **AND** more than 2 agents?

Readiness for physical therapy does **NOT** indicate specific treatment content or mobilization

If patient deemed not ready for physical therapy: Readiness re-assessed at least daily by PT and RN. Contact physical therapist if:

- Status changes within shift
- Positioning or range of motion concerns
- Other questions or concerns

Supplementary Material 3. Descriptive Statistics of Clinical-Operational Aims[a]

	**Pre**<br>*n = 5924*<br><br>*Aug 2011 – March 2015*	**During**<br>*n = 1389*<br><br>*April 2015 – Dec 2015*	**Post**<br>*n = 5745*<br><br>*Jan 2016 – May 2019*
**% of admits receiving physical therapy**	29%	67%	60%
**Hours from MICU admit to first physical therapy session**	55 (24-114)	23 (16-40)	28 (17-49)
**% of MICU days with physical therapy session**	33% (20-50)	67% (50-100)	56% (40-100)

[a]Percentage of days with physical therapist is expressed as a median and interquartile range (Q1, Q3) for patients who received physical therapy. Note that time-to-physical therapy is conditional on receiving physical therapy and thus shorter than the cohort-level median time-to-event in Figure 1. MICU = Medical ICU

Supplementary Material 4. Quantile Regression Model for Adjusted Effects of the Intervention Period (Relative to Pre-Intervention) on ICU Length of Stay (LOS) Across Various Quantiles of ICU LOS [a]

Quantile	Value (Hours)	During-Intervention Estimate (CI)	Post-Intervention Estimate (CI)
0.2	22	−1 (−3.6 to 3.5)	−0.5 (−4.1 to 1.3)
0.3	28	-1 (−3.7 to 0.6)	-1 (−3.7 to 0.4)
0.4	38	−2.5 (−7.1 to 2.1)	-2.5 (−5.6 to 1.1)
0.5	47	−4 (−9.2 to −0.8)	−4 (−7.6 to 2.4)
0.6	62	-4 (−11.7 to −0.3)	-5 (−9.1 to 1)
0.7	81	-6 (−13.7 to −1.3)	−7 (−12.7 to −1.8)
0.8	118	-9 (−19.4 to 1.5)	−9 (−21.6 to 3.5)
0.9	207	-14 (−24.3 to 2.1)	−16 (−32.1 to −3.6)

[a]ICU = intensive care unit.
